# Impact of Acute Eccentric versus Concentric Running on Exercise-Induced Fat Oxidation and Postexercise Physical Activity in Untrained Men

**DOI:** 10.1155/2020/2608730

**Published:** 2020-07-20

**Authors:** Shaea Alkahtani, Osama Aljuhani, Nasser Alkhalidi, Naif Almasuod, Omar Hezam, Ibrahim Aljaloud, Haitham Abdel Hamid Dawoud, Ahmed Abdusalam

**Affiliations:** ^1^Department of Exercise Physiology, College of Sport Sciences and Physical Activity, King Saud University, Riyadh, Saudi Arabia; ^2^Department of Physical Education, College of Sport Sciences and Physical Activity, King Saud University, Riyadh, Saudi Arabia; ^3^Department of Sport Health Sciences, Faculty of Physical Education, Helwan University, Cairo, Egypt; ^4^Department of Biomechanics and Motor Behavior, College of Sport Sciences and Physical Activity, King Saud University, Riyadh, Saudi Arabia

## Abstract

**Introduction:**

This study aimed at comparing the rate of exercise-induced fat oxidation and postexercise free-living physical activity after constant-load flat running (FR) and downhill running (DHR) bouts at an intensity that elicited maximal fat oxidation.

**Methods:**

Participants were 11 healthy untrained men (mean age 25.6 ± 3.3 years; VO_2max_39.11 ± 8.05 ml/kg/min). The study included four visits. The first two visits determined the intensity of maximal fat oxidation during incremental FR and DHR tests. The second two visits involved constant-load FR or DHR at the intensity that elicited maximal fat oxidation in a counterbalanced order separated by two weeks. Gas exchange analysis was used to measure substrate oxidation during all exercise sessions. Sedentary time and physical activity were measured using ActiGraph triaxial accelerometers for three days including the day of exercise tests (the second day).

**Results:**

During the incremental exercise tests, fat oxidation was significantly greater during the first stage of FR (*P* < 0.05) but started to increase during the fourth stage of DHR, although this did not reach significance. Of the 11 participants, 7 had greater fat oxidation during DHR. During continuous constant-load running, fat oxidation was higher during DHR than FR but at only two stages was either significant or borderline significant, and the time/group interaction was not significant. There was no significant effect on sedentary time of time/group interaction (*P* = 0.769), but there was a significant effect of time (*P* = 0.005), and there was no significant effect on total physical activity of time/group interaction (*P* = 0.283) or time (*P* = 0.602).

**Conclusion:**

Acute aerobic eccentric exercise at an intensity eliciting maximal fat oxidation enhanced exercise-induced fat oxidation without worsening postexercise free-living physical activity, indicating it could be a useful training modality in weight management programs.

## 1. Introduction

Eccentric exercise such as downhill running requires less metabolic effort than traditional running (i.e., flat running). This type of exercise is therefore a promising training strategy for a nonactive population such as obese and sedentary individuals. For example, in a study involving obese women, 12 weeks of descending stair walking improved functional fitness and blood profile to a greater extent than did ascending stair walking [1]. Likewise, Julian et al. [2] found that 12-week eccentric cycling significantly improved insulin resistance while concentric cycling did not, although both types of training improved maximal oxygen consumption, body composition, and leg strength. Eccentric exercise can improve metabolic and physical characteristics with fewer training sessions; for example, one session a week of eccentric isokinetic contraction for eight weeks in untrained individuals increased resting energy expenditure and fat oxidation [3].

However, there are methodological issues surrounding studies measuring internal physiological effort using energy expenditure and substrate oxidation and external work load using speed and resistance during eccentric exercise, which require further experimental research. For example, Penailillo et al. [4] found that fat oxidation during acute eccentric cycling was 72% greater than concentric cycling, and energy expenditure was 36% lower than concentric cycling. However, this experimental study used similar relative oxygen consumption, meaning different absolute power output. In another study, when the relative exercise intensity was kept constant at 60% VO_2peak_, there was no significant difference in fat oxidation between continuous 40-minute flat and downhill running bouts [5]. As fat oxidation is minimal at high-intensity exercise, the high intensity used in this study could explain the lack of difference between the conditions. Isacco et al. [6] compared concentric and eccentric cycling at 35% Vo_2peak_ for 45 min and found no significant differences in fat or carbohydrate (CHO) oxidation between the two conditions. However, it was not clear whether this intensity induced the greatest capacity for fat oxidation in both conditions. To examine this, a comparison study at the intensity that elicits maximal fat oxidation is required.

It is well established that moderate exercise intensity causes greater use of fat sources than higher intensity levels [7], and an optimal intensity that elicits maximal fat oxidation has been determined [8]. The level of fat oxidation in response to this optimal intensity differs according to several factors including mode, modality, gender, and fitness levels [9, 10] and has not been examined during eccentric exercise. Thus, the main aim of the present study was to compare the response of substrate oxidation at an intensity that elicits maximal fat oxidation during acute eccentric running compared to concentric flat running in healthy untrained men.

Many studies of traditional concentric exercise have found no effect of structured concentric exercise training on nonexercise energy expenditure and/or physical activity. For example, habitual physical activity and energy expenditure were monitored for 16 days in male and female participants, with four exercise bouts that expended 500 kcal performed in the second half of the intervention, and this study concluded that the exercise program did not change energy expenditure due to spontaneous activity [11]. A 2014 review concluded that there was limited evidence to support the hypothesis that prescribed exercise decreases nonexercise physical activity and energy expenditure, although proper techniques such as indirect calorimetry and doubly labeled water are required [12]. Unlike concentric traditional exercise, unaccustomed eccentric contractions produce muscle damage, soreness, and force impairment [13] that can last several days [14]. Such muscle soreness and damage decreases performance in strength and power tests in posteccentric cycling exercise [15]. The magnitude of muscle damage is affected by several factors including the intensity and slope of eccentric exercise [16]. We hypothesized that the intensity currently proposed to induce maximal fat oxidation causes low to mild muscle damage, such that it can increase exercise-induced fat oxidation with no impairment of postexercise free-living physical activity.

## 2. Methods

### 2.1. Participant Characteristics

The included participants were 11 healthy young men (mean age 25.6 ± 3.3 years; body mass index (BMI) 24.70 ± 2.14 kg/m^2^; VO_2max_39.11 ± 8.05 ml/kg/min; and maximal heart rate 191 ± 9 beats/min), who upon recruitment engaged in aerobic exercise for 30 to 150 minutes per week. This included recreational walking activities and job type (e.g., students of College of Sport Sciences and Physical Activity at King Saud University). Exclusion criteria included engagement in regular resistance training, BMI > 30 kg/m^2^, sedentary behavior (i.e., did not perform any training in a typical week), or engagement in regular aerobic training for more than 150 minutes per week.

### 2.2. Study Procedure

The experiment had a crossover counterbalanced design separated by two weeks and took place at the Exercise Physiology Laboratories at the Exercise Physiology Department, College of Sport Sciences and Physical Activity, King Saud University (KSU). The laboratory is air-conditioned, with the temperature held constant at 21°C. The study included four visits: two to determine the intensity at which maximal fat oxidation was induced during concentric exercise (flat running) and eccentric exercise (downhill running). The second two visits involved continuous flat and downhill running at the intensity that elicited maximal fat oxidation. Gas exchange analysis was used to measure the substrate oxidation during all exercise sessions, as described below.

All participants were asked to maintain their normal dietary intake between tests and to replicate their average food intake as closely as possible on the day before the exercise tests. Participants were asked to abstain from strenuous exercise and excessive consumption of caffeine in the 24 h before the test. All participants were instructed to arrive at the laboratory between 8 am and 11 am, following an overnight fasting. The experiment was conducted with the human subjects' understanding and consent of the experimental process The Institutional Review Board (IRB) at KSU approved the study procedure (reference number KSU-SE-17-6).

### 2.3. Incremental and Constant-Load Exercise Tests

A graded exercise test was performed on a treadmill beginning at 4 km/h with a 0% gradient for 3 minutes per stage, with the speed increasing by 1 km/h per stage until the respiratory exchange ratio (RER) reached 1.0, in order to determine the intensity that elicited maximal fat oxidation. Maximal aerobic capacity was measured during the flat running (FR) test only, where participants rested for 10 minutes after the first stage and resumed running at a gradient of 3.5%, which increased by 2.5% every three minutes until volitional exhaustion was reached.

To determine the intensity that elicits maximal fat oxidation during downhill running (DHR), a graded exercise test was performed on a treadmill beginning at 4 km/h with a –12% gradient for 3 minutes per stage, with the speed increasing by 1 km/h per stage until the RER reached 1.0.

Two running constant-load exercise tests were performed on separate days at gradients of 0% (FR) and –12% (DHR) for 40 minutes, consisting of 8 minutes of running at the intensity that elicited maximal fat oxidation during incremental FR and DHR interspersed by low-intensity walking for 2 minutes.

### 2.4. Measurement of Substrate Oxidation

Gas exchange was collected and analyzed during all exercise tests using the Parvo Medics Analyser Module (TrueOne® 2400, Metabolic Measurement System, Parvo Medics Inc., USA), which was calibrated for gas and flow meter before all tests following the manufacturer's guidelines. Participants also wore a Polar heart rate (HR) chest strap to monitor exercise-induced HR (Polar H10, Polar Electro 2020, NY, USA).

Expired air measurements were averaged every 30 seconds, and consumption of oxygen (VO_2_) and carbon dioxide (VCO_2_) in liters per minute and RER were exported to an Excel file. Fat and CHO oxidation were calculated using the following formulae:

(1) $$\begin{array}{c} \text{Total fat oxidation}=1.67\text{ V}{\text{O}}_{2}–1.67\text{ VC}{\text{O}}_{2},\\ \text{Total CHO oxidation}=4.55\text{ VC}{\text{O}}_{2}–3.21\text{ V}{\text{O}}_{2}. \end{array}$$

### 2.5. Measurement of Sedentary Time and Physical Activity

The participants' sedentary time and physical activity were measured using ActiGraph triaxial accelerometers (wGT3X-BT, ActiGraph LLC, Pensacola, FL). Accelerometers were initialized, and then, data were downloaded and analyzed using ActiLife v6013.3 (ActiGraph LLC, Pensacola, FL). The participants were instructed to wear their accelerometer for three days, including the day of the exercise test (the second day). Participants were instructed to wear the accelerometers on their right hip at all times except when they were bathing or sleeping.

The raw data from the accelerometers were downloaded, and counts per minute (CPM) were calculated from the vertical axis movement. The wear time of the accelerometers was validated using the algorithm published by Troiano et al. [17]. Nonwear time was classified as a zero reading sustained for a period of 60 minutes with a tolerance of 1–2 minutes of counts between 0 and 100. Participants with valid data of a minimum of 600 minutes of wear time on all three days were included in the final analysis.

Cut-points developed by Freedson et al. [18] were used to categorize physical activity intensities as sedentary (<99 CPM), light activity (100–1951 CPM), moderate activity (1952–5724 CPM), vigorous activity (5725–9498 CPM), and very vigorous activity (≥9499 CPM).

### 2.6. Statistical Analysis

Data were analyzed using Statistical Package for the Social Sciences (SPSS) version 25 for Windows (SPSS Statistics 25.0, IBM, NY, USA). Two-way repeated-measures tests were performed to analyze the exercise stages (five stages in each session) and exercise conditions (FR and DHR) for oxygen consumption and substrate oxidation. Two-way repeated-measures tests were also performed to analyze the exercise conditions (FR and DHR) and time of measurements (pre- and postexercise) for sedentary and total physical activity. Independent sample *t*-tests were conducted to evaluate significant differences between FR and DHR at each comparable time point of all tests. An *α*-level of 0.05 was used to determine statistical significance.

## 3. Results

The incremental running test showed significantly higher levels of oxygen consumption during FR than DHR at all stages from 4 to 8 km/h (*P* < 0.001), as shown in Table 1.

**Figure 1:**
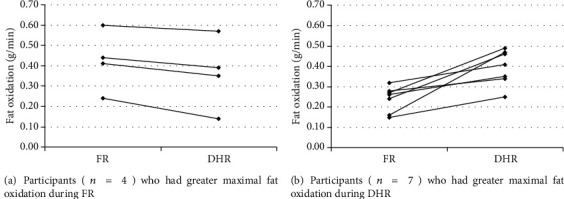
Individual variation in the maximal fat oxidation (a, b) during incremental exercise tests of flat running (FR) and downhill running (DHR).

Fat oxidation was significantly greater during FR in the first stage (*P* < 0.05), while it started to increase from the fourth stage during DHR but did not reach significance. In the last two stages, RER was significantly higher during FR than during DHR (*P* < 0.05).  Figure 1 shows the individual variation in the maximal fat oxidation during the incremental test of FR and DHR; 4 out of 11 participants had greater fat oxidation during FR.

During continuous constant-load running, there were no significant differences in VO_2_ between FR and DHR, although the mean value of VO_2_ was greater at all stages during DHR, leading to a significant time effect and time∗group effect, as shown in Table 2.

CHO oxidation was higher during FR than during DHR in four stages, but the differences were not significant. The fat oxidation was higher during DHR than during FR but at only two stages was this difference either significant or borderline significant, and time∗group was not significant. The average contribution of fat and CHO sources to energy expenditure was 38.3 ± 2.8% and 61.7 ± 2.8%, respectively, for FR and 52.0 ± 1.2% and 48.0 ± 1.2%, respectively, for DHR.

In terms of sedentary activity, there was no significant effect of time∗group (*P* = 0.769), and there was a significant effect of time (*P* = 0.005). Time spent in sedentary activity was higher before DHR than before FR by 74.2 min (95% confidence interval (CI) 4.6, 143.9; *t* [10] = 2.376; and *P* = 0.039) and after DHR than after FR by 90.4 min (95% CI 3.5, 177.3; *t* [10] = 2.319; and *P* = 0.043, Table 3).

For total physical activity, there was no significant effect of time∗group (*P* = 0.283), and there was no significant effect of time (*P* = 0.602). Time spent in total physical activity was higher after FR than after DHR by 72.7 min (95% CI 3.90, 141.5; *t* [10] = 2.355; and *P* = 0.040, Table 3).

## 4. Discussion

This study aimed at comparing the maximal fat oxidation during incremental FR and DHR tests and the rate of exercise-induced fat oxidation and postexercise free-living physical activity of constant-load FR and DHR exercise at the point of maximal fat oxidation. During the incremental exercise test, the response of fat oxidation at a matched running speed was higher during FR than during DHR at the first two stages but was higher during the later stages of DHR, although this difference was not significant. The maximal fat oxidation was greater during DHR in 7 out of 11 participants. The fat oxidation rate during exercise sustained at an intensity that elicited maximal fat oxidation was greater during DHR than during FR. Moreover, this intensity level of DHR did not affect postexercise free-living physical activity, which may suggest that it induced no or mild muscle damage.

The study participants were untrained young Saudi men, and their maximal fat oxidation was low, comparable with previously published sedentary, type 2 diabetes, and obese groups. For example, the maximal fat oxidation during FR and DHR in this study was close to or comparable with women with type 2 diabetes (0.38 g/min), and fat oxidation during FR and DHR was lower than untrained healthy women (0.54 g/min) [19]. The same outcomes were found in obese young men (0.38 ± 0.13 g/min) while fat oxidation in the active was 0.58 ± 0.07 g/min [20]. The low level of fat oxidation in the present healthy young male participants raises the question of whether ethnicity may [21] or may not [22, 23] partially explain variations of fat oxidation; further studies on Saudi and Arab populations are required to answer this.

In the constant-load exercise bouts, the increase in VO_2_ was greater during DHR than during FR, which could be attributed to the increase of the VO_2_ slow component due to greater fatigue on motor units during eccentric contraction forcing recruitment of additional motor units to achieve the same work rate [24]. This increase in mechanical demands during DHR was accompanied by an increase in HR. These mechanical demands of eccentric muscle contraction did not decelerate the response of fat oxidation with the constant-load exercise, but the muscles filled energy demands using CHO sources rather than using fat sources. We previously found that the alteration in exercise-induced energy demands during concentric moderate-intensity interval exercise is largely filled by CHO sources with no effect on the gradually constant increase in fat oxidation [25]. The rate of fat oxidation increased with exercise time (significant time effect), independent of exercise modality (insignificant time∗group effect), which is in line with the concept that fat is oxidized during constant-load exercise [26, 27].

The contribution of the fat source to energy expenditure during both conditions was not affected by time, remaining constant during both FR and DHR. Isacco et al. [6] found that the contribution of CHO and fat source in energy expenditure was 53.1% CHO and 46.9% fat during concentric exercise vs. 46.2% CHO and 53.8% fat during eccentric exercise. In our study, the contributions of CHO and fat sources to energy expenditure were 61.7% and 38.3%, respectively, during FR vs. 48.0% and 52.0%, respectively, during DHR. It should be noted that Isacco et al. [6] used a bicycle at 30 revolutions per minute, with the same relative intensity during concentric and eccentric exercises, but this does not explain the differences to our study in the concentric modality results but comparable eccentric modality results. Penailillo et al. [4] conducted exercise bouts at 60% of maximal concentric power output (relative intensity: 76% for concentric and 38% for eccentric) and found that both energy expenditure and CHO expenditure were lower during eccentric than concentric exercise by 36% and 42%, respectively, and fat utilization was greater during eccentric than concentric by 72%. This difference between concentric and eccentric exercises is greater than in the present study or in Isacco et al.'s study.

Several confounders should be considered when comparing the present concentric and eccentric outcomes with other available studies. For example, the –12% gradient used in the present study could increase cardiovascular and metabolic demands and muscle damage to a greater extent than gentler inclinations, such as between –5% and –10% [28]. Moreover, several factors such as insulin level [19], mode of exercise [29], and the recruitment of muscle mass during the test [30] influence the rate of exercise-induced fat oxidation. For example, when individuals performed cycling and rowing exercises at the same intensity, fat oxidation was greater during the rowing session, and this was likely due to the larger muscle mass recruited during rowing [30]. Lastly, Penailillo et al. [4] reported that all participants showed greater fat oxidation during eccentric exercise, while four of our study participants had a greater rate of fat oxidation during the concentric FR exercise (Figure 1). Large individual variations in the rate of maximal fat oxidation have been previously reported [31].

Considering the pre- and postexercise activity levels, our data showed that there were no significant interactions between exercise duration and modality and no statistically significant differences between pre- and postexercise in time spent in sedentary and physical activity in both exercise conditions. The small reduction in total physical activity after DHR (–4.7%) could be attributed to mild muscle damage, while physical activity after FR tended to increase (6.9%). This finding is in line with some previous studies in supporting the importance of low-intensity eccentric exercise in general population exercise programs. For example, downhill walking at 6 km/h and an inclination of –5% for 30 min caused mild muscle damage and delayed-onset muscle soreness the day after exercise compared with uphill walking at an inclination of 5%, but this did not cause significant impairment in glucose metabolism [32]. Likewise, low to mild muscle damage after eccentric exercise did not alter postexercise metabolic responses (i.e., glucose tolerance) [33]. Our results prove that performing eccentric exercise at an intensity that elicits maximal fat oxidation (i.e., speed at 7 to 8 km/h with an inclination of –12%) did not affect postexercise free-living physical activity.

It is known that an initial bout of acute eccentric exercise can cause increased postexercise muscle damage, which can be gradually relieved with subsequent repeated exercise bouts. However, the magnitude of impact during acute eccentric exercise and the local muscle adaptation during chronic eccentric training among heterogeneous individuals is not conclusively known. For example, in a study of active young men conducted in our laboratory, half of the participants showed no differences in voluntary muscle contraction capability between pre-, post-, and 24 h postacute eccentric exercise, demonstrating that the impact of acute eccentric exercise on muscle damage was minimal in this population [5]. Melanson et al. [34] stated that there are wide variations among individuals in their nonexercise physical activity response to structured exercise training.

## 5. Conclusion

With the qualification that not all participants had a greater rate of fat oxidation during incremental eccentric than concentric exercise bout, this study showed that acute aerobic eccentric exercise at an intensity eliciting maximal fat oxidation enhanced exercise-induced fat oxidation without worsening postexercise free-living physical activity, indicating it could be a useful training modality in weight management programs.

Future studies should investigate whether any expected improvement in body weight during DHR is due to the greater utilization of fat or due to the higher amount of work load compared to FR. Moreover, as the responses of VO_2_ and HR during DHR were different from FR, and due to the fact that monitoring fat oxidation is not feasible during filed exercise training, understanding the adaptation of physiological parameters at the level of maximal fat oxidation should be examined in laboratory and field chronic experimental studies.

## Figures and Tables

**Table 1:** Comparison between physiological responses during incremental flat running (FR) and downhill running (DHR) tests.

Speed	FR	DHR	Mean change (95% CI)	Percentage change (%)	*P* value
VO_2_ (ml/min/kg)					
4 km/h	9.35 ± 0.6	7.34 ± 0.7	2.01 (1.6-2.4)	21.45	<0.001
5 km/h	11.20 ± 0.7	8.18 ± 0.8	2.97 (2.3-3.7)	26.52	<0.001
6 km/h	14.84 ± 2.4	9.87 ± 0.9	4.98 (3.5-6.5)	33.56	<0.001
7 km/h	20.80 ± 2.2	15.84 ± 1.7	4.92 (3.7-6.2)	23.65	<0.001
8 km/h	25.34 ± 2.2	19.82 ± 1.5	5.51 (3.5-7.5)	21.74	<0.001
Time effect	<0.001				
Time∗group	<0.001				

Fat oxidation (g/min)					
4 km/h	0.20 ± 0.08	0.10 ± 0.11	0.10 (0.04-0.17)	50	0.033
5 km/h	0.25 ± 0.11	0.16 ± 0.10	0.09 (0.009-0.18)	36	0.051
6 km/h	0.27 ± 0.15	0.20 ± 0.10	0.07 (-0.02-0.16)	25.9	0.231
7 km/h	0.22 ± 0.92	0.34 ± 0.13	-0.12 (-0.06-0.25)	54.4	0.186
8 km/h	0.16 ± 0.17	0.34 ± 0.14	-0.17 (-0.38-0.03)	106.3	0.071
Time effect	0.535				
Time∗group	0.060				

CHO oxidation (g/min)					
4 km/h	0.46 ± 0.20	0.46 ± 0.31	0.003 (-0.21-0.21)	0.65	0.978
5 km/h	0.52 ± 0.24	0.38 ± 0.21	0.14 (-0.10-0.38)	26.92	0.180
6 km/h	0.89 ± 0.33	0.41 ± 0.22	0.47 (0.20-0.75)	52.81	0.001
7 km/h	1.71 ± 0.78	0.65 ± 0.31	1.06 (0.43-1.69)	61.99	0.034
8 km/h	2.05 ± 0.92	0.99 ± 0.42	1.06 (0.32-1.79)	51.71	0.012
Time effect	<0.001				
Time∗group	0.020				

RER					
4 km/h	0.84 ± 0.05	0.89 ± 0.12	-0.05 (-0.12-0.03)	5.95	0.294
5 km/h	0.84 ± 0.06	0.84 ± 0.07	-0.01 (-0.07-0.06)	1.19	0.820
6 km/h	0.87 ± 0.05	0.83 ± 0.07	0.04 (-0.01-0.10)	4.59	0.192
7 km/h	0.92 ± 0.07	0.83 ± 0.06	0.09 (0.03-0.15)	9.78	0.007
8 km/h	0.94 ± 0.07	0.86 ± 0.06	0.08 (0.01-0.15)	8.51	0.034
Time effect	<0.001				
Time∗group	0.004				

Data are presented as mean ± SD. FR: flat running; DHR: downhill running; CI: confidence interval; CHO: carbohydrate; RER: respiratory exchange ratio. *P* value significant at *P* < 0.05, 0.01 level.

**Table 2:** Comparison between physiological responses during constant-load flat running (FR) and downhill running (DHR) tests.

Time	FR	DHR	Mean change (95% CI)	Percentage change (%)	*P* value
VO_2_ (ml/min/kg)					
8 minutes	15.48 ± 5.6	17.91 ± 4.8	-2.43 (-7.7-2.8)	15.69	0.413
18 minutes	15.21 ± 5.9	17.91 ± 4.8	-2.70 (-7.8-2.4)	17.75	0.273
28 minutes	15.32 ± 5.5	18.73 ± 5.5	-3.4 (-8.5-1.7)	22.19	0.184
38 minutes	15.42 ± 5.34	19.27 ± 5.8	-3.86 (-9.0-1.3)	25.03	0.139
48 minutes	15.89 ± 5.6	19.42 ± 5.8	-3.52 (-8.6-1.6)	22.15	0.186
Time effect	<0.001				
Time∗group	0.006				

Fat oxidation (g/min)					
8 minutes	0.26 ± 0.15	0.38 ± 0.19	-0.13 (-0.25–-0.004)	50.0	0.120
18 minutes	0.22 ± 0.12	0.37 ± 0.21	-0.15 (-0.26–-0.04)	68.18	0.056
28 minutes	0.23 ± 0.13	0.41 ± 0.24	-0.18 (-0.32–-0.05)	78.26	0.046
38 minutes	0.25 ± 0.15	0.41 ± 0.24	-0.16 (-0.30–-0.02)	64.0	0.089
48 minutes	0.28 ± 0.14	0.42 ± 0.25	-0.14 (-0.28-0.01)	50	0.146
Time effect	0.016				
Time∗group	0.319				

CHO oxidation (g/min)					
8 minutes	0.91 ± 0.43	0.76 ± 0.28	0.14 (-0.24-0.52)	15.38	0.384
18 minutes	0.97 ± 0.45	0.85 ± 0.36	0.12 (-0.33-0.57)	12.37	0.532
28 minutes	0.97 ± 0.49	0.83 ± 0.36	0.14 (-0.31-0.58)	14.43	0.487
38 minutes	0.91 ± 0.45	0.87 ± 0.36	0.04 (-0.40-0.48)	4.39	0.833
48 minutes	0.88 ± 0.43	0.88 ± 0.40	-0.01 (-0.43-0.42)	1.13	0.971
Time effect	0.420				
Time∗group	0.300				

RER					
8 minutes	0.87 ± 0.06	0.83 ± 0.04	0.04 (-0.003-0.08)	4.59	0.113
18 minutes	0.89 ± 0.05	0.84 ± 0.05	0.05 (0.01-0.09)	5.62	0.041
28 minutes	0.89 ± 0.06	0.83 ± 0.04	0.05 (0.01-0.10)	5.62	0.036
38 minutes	0.88 ± 0.06	0.84 ± 0.04	0.04 (-0.01-0.08)	4.54	0.150
48 minutes	0.86 ± 0.06	0.84 ± 0.05	0.03 (-0.02-0.08)	3.49	0.274
Time effect	0.212				
Time∗group	0.323				

HR (beat/min)					
8 minutes	112.3 ± 21.56	126.8 ± 22.50	-14.5 (-34.9-5.9)	12.91	0.158
18 minutes	112.80 ± 17.45	131.60 ± 21.87	-18.8 (-37.8-0.22)	16.67	0.048
28 minutes	117.40 ± 16.83	136.80 ± 25.34	-19.40 (-38.9-0.15)	16.52	0.059
38 minutes	115.3 ± 15.73	138.70 ± 28.14	-23.4 (-46.6–-0.22)	20.29	0.034
48 minutes	115.7 ± 22.25	141.80 ± 27.61	-26.1 (-52.9-0.68)	22.56	0.032
Time effect	<0.001				
Time∗group	0.053				

Data are presented as mean ± SD. FR: flat running; DHR: downhill running; CI: confidence interval; CHO: carbohydrate; RER: respiratory exchange ratio; HR: heart rate. *P* value significant at *P* < 0.05, 0.01 level.

**Table 3:** Time spent in sedentary and physical activity pre- and postexercise.

Variables	FR (mean ± SD)	DHR (mean ± SD)
Sedentary (minutes per day)		
Preexercise	702.5 ± 116.6	776.8 ± 76.3^∗^
Postexercise	693.8 ± 70.9	784.2 ± 120.5^∗^
Total physical activity (minutes per day)		
Preexercise	260.4 ± 86.3	235.6 ± 95.9
Postexercise	279.7 ± 96.8	225.0 ± 131.7^∗^

Data are presented as mean ± SD. FR: flat running; DHR: downhill running. ^∗^Significantly different (*P* ≤ 0.05) compared to FR.

## Data Availability

The data used to support the findings of this study are included within the article. For further information, please contact the corresponding author.
